# The Single Particles, Clusters and Biomolecules and Serial Femtosecond Crystallography instrument of the European XFEL: initial installation^1^

**DOI:** 10.1107/S1600577519003308

**Published:** 2019-04-12

**Authors:** Adrian P. Mancuso, Andrew Aquila, Lewis Batchelor, Richard J. Bean, Johan Bielecki, Gannon Borchers, Katerina Doerner, Klaus Giewekemeyer, Rita Graceffa, Oliver D. Kelsey, Yoonhee Kim, Henry J. Kirkwood, Alexis Legrand, Romain Letrun, Bradley Manning, Luis Lopez Morillo, Marc Messerschmidt, Grant Mills, Steffen Raabe, Nadja Reimers, Adam Round, Tokushi Sato, Joachim Schulz, Cedric Signe Takem, Marcin Sikorski, Stephan Stern, Prasad Thute, Patrik Vagovič, Britta Weinhausen, Thomas Tschentscher

**Affiliations:** a European XFEL, Holzkoppel 4, 22869 Schenefeld, Germany; b Department of Chemistry and Physics, La Trobe Institute for Molecular Science, La Trobe University, Melbourne, Victoria 3086, Australia; c Center for Free Electron Laser Science, Deutsches Elektronen-Synchrotron, Notkestraße 85, 22607 Hamburg, Germany

**Keywords:** XFEL, serial crystallography, instrumentation

## Abstract

The European X-ray Free-Electron Laser (FEL) became the first operational high-repetition-rate hard X-ray FEL with first lasing in May 2017. Biological structure determination has already benefitted from the unique properties and capabilities of X-ray FELs, predominantly through the development and application of serial crystallography. The possibility of now performing such experiments at data rates more than an order of magnitude greater than previous X-ray FELs enables not only a higher rate of discovery but also new classes of experiments previously not feasible at lower data rates. One example is time-resolved experiments requiring a higher number of time steps for interpretation, or structure determination from samples with low hit rates in conventional X-ray FEL serial crystallography. Following first lasing at the European XFEL, initial commissioning and operation occurred at two scientific instruments, one of which is the Single Particles, Clusters and Biomolecules and Serial Femtosecond Crystallography (SPB/SFX) instrument. This instrument provides a photon energy range, focal spot sizes and diagnostic tools necessary for structure determination of biological specimens. The instrumentation explicitly addresses serial crystallography and the developing single particle imaging method as well as other forward-scattering and diffraction techniques. This paper describes the major science cases of SPB/SFX and its initial instrumentation – in particular its optical systems, available sample delivery methods, 2D detectors, supporting optical laser systems and key diagnostic components. The present capabilities of the instrument will be reviewed and a brief outlook of its future capabilities is also described.

## Abstract 2

An introduction to the early operational capabilities of the Single Particles, Clusters and Biomolecules and Serial Femtosecond Crystallography (SPB/SFX) scientific instrument at the European X-ray Free Electron Laser facility is presented.

## 1. Introduction   

The recently operational European X-ray Free-Electron Laser (EuXFEL) is the first high-repetition-rate hard X-ray FEL in the world (Abela *et al.*, 2006 ▸). The initial suite of six scientific instruments is designed to pursue a wide range of science applications exploiting both the ultrafast and ultrabright pulses of X-ray FEL radiation produced, as well as uniquely utilizing the megahertz (MHz) peak repetition rate of this unprecedented facility (Tschentscher *et al.*, 2017 ▸). The Single Particles, Clusters and Biomolecules and Serial Femtosecond Crystallography (SPB/SFX) instrument is one of the first two instruments to come online and perform experiments with the EuXFEL beam and has produced first results less than a year after first experiments (Grünbein *et al.*, 2018 ▸; Wiedorn *et al.*, 2018 ▸).

The SPB/SFX instrument is primarily concerned with 3D structure determination of both crystalline and non-crystalline micrometre-scale and smaller objects. A particular emphasis is placed on biological objects, including viruses, biomolecules and protein crystals. Nevertheless, the instrument will also be capable of investigating non-biological samples using similar techniques. The instrument exploits the unique high peak repetition rate of the facility and brings benefit to these structural biology applications. The major benefits are the reduction in the experimental time required to measure a complete dataset for structure determination, particularly in time-resolved experiments and potential reduction in sample consumption in some methods.

The goal of this paper is to describe the capabilities of the SPB/SFX instrument in its early operational phase. Future capabilities, such as additional interaction regions for further experiments (such as in-atmosphere experiments) are also briefly outlined. The instrument relies on a variety of complex systems, many of which are described in more detail in this journal. These systems include the accelerator (Decking *et al.*, 2019 ▸), the photon transport systems (Sinn *et al.*, 2019 ▸) from EuXFEL’s undulator source (Abeghyan *et al.*, 2019 ▸) to the instrument and the detector systems (Henrich *et al.*, 2011 ▸); in addition, the optical laser systems (Palmer *et al.*, 2019 ▸) that provide optical pulses at the same repetition rate as the EuXFEL for so-called pump–probe experiments that excite (‘pump’) a system with an optical pulse and investigate (‘probe’) it with the X-ray FEL pulses (or vice versa). All of this activity is supported by the controls system (Heisen *et al.*, 2013 ▸; Hauf *et al.*, 2019 ▸) while the extreme data rate due to the high repetition rate is managed by a state-of-the-art data acquisition and storage system (Boukhelef *et al.*, 2013 ▸). An essential part of fully utilizing the instrument is the data analysis tools to make sense of the so-called ‘data deluge’ which, while essential to the successful realization of experiments, is beyond the scope of this paper. EuXFEL data analysis tools are discussed in general by Fangohr *et al.* (2018 ▸).

This paper primarily documents the initial configuration of the instrument as commissioned for early user experiments in late 2017 and 2018. Some outlook is given towards the additional instrumentation to be installed in 2019, in particular brief descriptions of the additional interaction regions contributed by the SFX user consortium (SFX User Consortium, 2013 ▸), which will both broaden the instrument capability and increase its capacity in the near future.

## 2. Science targets for the instrument   

The SPB/SFX instrument predominantly caters to structure determination of biological systems, both crystalline samples and single particles. Potentially, any sample that can be investigated by forward-scattering methods may be probed at SPB/SFX, with the major use cases outlined below. These use cases are very similar to those of the Coherent X-ray Imaging (CXI) instrument at LCLS (Boutet & Williams, 2010 ▸), which has pioneered this science at 120 Hz repetition rate.

### 2.1. Serial crystallography   

The relatively recent advent of structure determination from micrometre- and sub-micrometre-sized crystals of biomolecules using X-ray FEL sources (Chapman *et al.*, 2011 ▸; Aquila *et al.*, 2012 ▸; Boutet *et al.*, 2012 ▸; Redecke *et al.*, 2013 ▸; Liu *et al.*, 2013 ▸; Barends *et al.*, 2014 ▸) represents a significant broadening of the scope of crystallography for biological structure determination (Standfuss & Spence, 2017 ▸). Importantly, serial crystallography at FELs addresses three major classes of sample: (i) those that do not form large enough crystals to provide an adequate signal-to-noise ratio for analysis of their diffraction using a synchrotron source (Redecke *et al.*, 2013 ▸), (ii) those containing metal atoms that may be easily altered chemically by longer duration synchrotron radiation (and hence not reflect the native structure of the sample) (Suga *et al.*, 2014 ▸), and (iii) time-resolved systems, where either femtosecond time resolution is needed or, for example, irreversible reactions such as mixing which requires small crystals to minimize the mixing time and define a clear *t*_0_ as the start of the reaction (Schmidt, 2013 ▸).

### 2.2. Single particle imaging   

Arguably one of the most exciting classes of samples for structure determination at an X-ray FEL facility encompasses those samples where each individual sample exhibits the same, or markedly similar, structure as the others. A so-called ‘reproducible’ sample allows for the collection of data from many copies of such samples. Naively, each individual sample illuminated with the X-ray FEL beam scatters 2D data into a 2D detector at the instrument. Reproducible samples allow the aggregation of that data from many randomly oriented copies of the sample into a single piece of 3D information about the sample (Neutze *et al.*, 2000 ▸; Ekeberg *et al.*, 2015 ▸). This method is referred to as single particle imaging (SPI). The method is presently under development (Aquila *et al.*, 2015*a* ▸; Daurer *et al.*, 2017 ▸; Hantke *et al.*, 2018 ▸) and typically requires a large amount of diffraction data for successful three-dimensional reconstruction (Rose *et al.*, 2018 ▸; Lundholm *et al.*, 2018 ▸). The SPB/SFX instrument has the potential to collect SPI data almost 30 times faster than the X-ray FEL with the next highest repetition rate, potentially a significant step in this method’s development to application.

### 2.3. Time-resolved experiments   

One of the key advantages of X-ray FELs is in the ability to access the time domain on femtosecond timescales. This can be done either by exploiting the femtosecond pulse duration of an X-ray FEL to probe femtoscond timescale science, such as in the switching of magnetic structures (Beaurepaire *et al.*, 2004 ▸; Higley *et al.*, 2016 ▸), or by using an X-ray FEL’s extraordinary pulse intensities to allow the probing of irreproducible processes that require sufficient information to be acquired at each time point from a single measurement, such as mixing a protein with an antibiotic, or other mixing processes (Stagno *et al.*, 2016 ▸). The EuXFEL’s vastly greater repetition rate than other X-ray FEL sources is particularly attractive for time-resolved studies, as it allows for the collection of sufficient data from multiple time points within a realistic and manageable experiment duration. This then allows for a far greater number of time points to be explored, which is useful when changes in a sample occur over a variety of timescales or when one simply does not know the ideal timescale to make observations *a priori* and a survey in time is needed to establish the appropriate measurement parameters.

In addition to mixing experiments (Schmidt, 2013 ▸; Stagno *et al.*, 2016 ▸), reactions in bio-systems started by optical pulses are the other most common type of time-resolved experiment considered here. Pump–probe experiments excite a sample by an optical ‘pump’, causing changes to its structure. The subsequent X-ray FEL ‘probe’ is used to observe its structure some defined time after the excitation. Doing so for a number of different delay times between pump and probe allows for a so-called ‘molecular movie’ of the structural changes to be made (Tenboer *et al.*, 2014 ▸; Barends *et al.*, 2015 ▸; Levantino *et al.*, 2015*a* ▸; Pande *et al.*, 2016 ▸; Nango *et al.*, 2016 ▸), giving insights into the dynamics of such light-activated systems.

### 2.4. Further science goals   

Additional science cases include those supported by other classes of forward-scattering experiments, such as time-resolved small-angle X-ray scattering (Graceffa *et al.*, 2013 ▸; Levantino *et al.*, 2015*b* ▸; Bruetzel *et al.*, 2018 ▸; Röllen *et al.*, 2018 ▸) and X-ray photon correlation spectroscopy (XPCS) (Carnis *et al.*, 2014 ▸; Lehmkühler *et al.*, 2015 ▸).

## 3. Science requirements   

The science requirements of the instrument are derived directly from the science cases above and have not changed markedly since their publication in the SPB Technical Design Report (TDR) (Mancuso *et al.*, 2013 ▸). Importantly, the sample–detector distance must be compatible with the resolution and sampling requirements of samples of the order of less than 100 nm to approximately 1 µm in size [typical protein and/or protein (micro-/nano-)crystal sizes] (The AGIPD Consortium, 2012 ▸; Giewekemeyer *et al.*, 2013 ▸). The 2D detector used must have a very high dynamic range to accommodate not only the brightest signals from crystal Bragg peaks but also low signal on a single photon level, *e.g.* from the continuous diffraction between Bragg peaks (Ayyer *et al.*, 2016 ▸), which may fall into adjacent pixels in any practical detector. Single-photon sensitivity is also essential for SPI applications where a weakly scattering non-crystalline sample is envisaged and only a few photons per frame may be collected by the 2D detector (Loh & Elser, 2009 ▸; Ayyer *et al.*, 2014 ▸).

The X-ray focal spot size must also match these size ranges to optimally illuminate these smaller and larger samples. In practice, a variation of focal spot sizes at the sample position differing by an order of magnitude in lateral extent is desirable, realized at SPB/SFX by two independent focusing mirror systems.

Updated instrument parameters are shown in Table 1 ▸. While there have been a great deal of developments in serial crystallography and single particle imaging since the TDR (Mancuso *et al.*, 2013 ▸) was written, only relatively minor updates to the target parameters have been necessary.

## 4. Instrumentation   

**Figure 1:**
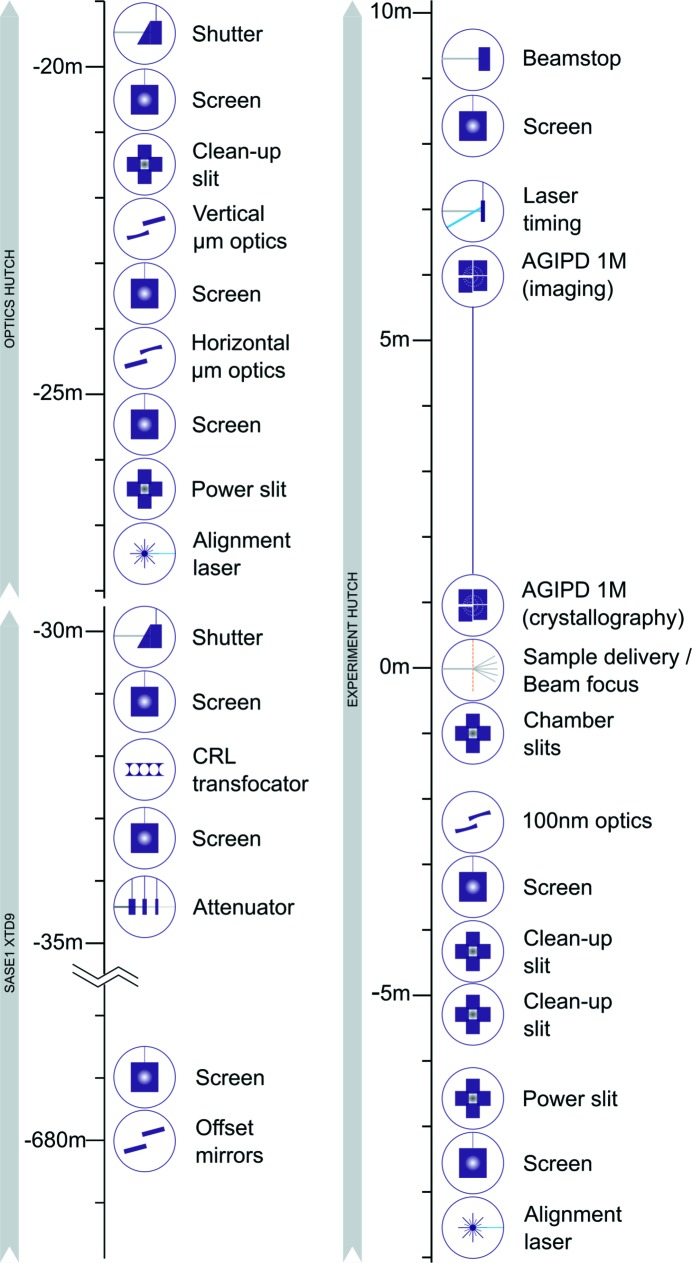
Iconographic layout of components of the SPB/SFX instrument. Approximate distances of components in the SASE1 XTD9 tunnel, SPB/SFX optics hutch and SPB/SFX experiment hutch are given relative to the common focal plane of the upstream interaction region. X-ray beam direction is indicated by the grey arrows.

The SPB/SFX instrument layout and instrumentation have been designed to accommodate experiments that lie within the main science goals while maintaining flexibility for future upgrades and alternative experiment types (Mancuso *et al.*, 2013 ▸). SPB/SFX is a forward-scattering instrument, with multiple optical systems to focus the FEL beam, a flexible sample–X-ray beam interaction environment with integrated sample-pump laser system, and an X-ray detector that is designed to collect scattering around the incident beam axis. These principal components are complemented by an array of X-ray beam conditioning and diagnostic devices. An iconographic overview of the instrument with the currently installed instrumentation is shown in Fig. 1 ▸.

**Figure 2:**
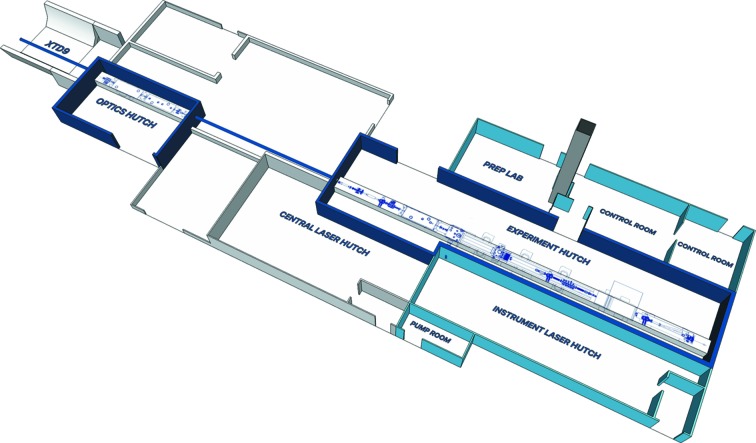
Overview of SASE1 hutches. X-ray beam direction is left to right, top to bottom. SPB/SFX X-ray radiation hutches are depicted in dark blue with SPB/SFX laser hutches, laboratory, control rooms and service rooms depicted in light blue. The vertical column in grey is the so-called ‘sample elevator’ used to transport material from the upstairs biological laboratory to the preparation laboratory adjacent to the SPB/SFX experiment hutch. A 2D overhead engineering view of the instrumentation is placed into the hutch model for context.

The SPB/SFX instrument is designed to operate at photon energies from 3 to 16 keV, with peak performance expected between about 6 and 15 keV. The SPB/SFX instrument is located behind the SASE1 undulator, with X-ray beam delivered via a dedicated branch in the XTD9 photon tunnel (Sinn *et al.*, 2019 ▸). Instrumentation is installed in the XTD9 tunnel, an instrument optics hutch and an experiment hutch. The geographical layout of SPB/SFX, including the relevant tunnel and hutches, is shown in Fig. 2 ▸.

The most upstream components of the SPB/SFX instrument – train picker, attenuator array and incoming beam diagnostic screen – are housed in the last metres of the SPB/SFX photon branch line in the XTD9 tunnel, upstream of the instrument shutter. A compound refractive lens system, used to provide focusing prior to the commissioning of the mirror-based focusing systems, was also installed at this location.

Directly following the tunnel downstream wall, the optics hutch houses a reference laser system (also sometimes called an alignment laser system), an aperture-defining B_4_C blade slit system, two vacuum chambers containing mirror optics of the micrometre-scale KB focusing system, clean-up slits to remove scatter around the beam, and a shutter to control transmission of the X-ray beam to the experiment hutch. Three diagnostic screens are located at the upstream, centre and downstream ends of the mirror chambers to aid mirror alignment.

Following ∼12 m of beam transport, the most upstream component in the SPB/SFX experiment hutch is a second reference laser system followed by a diagnostic screen and a second aperture-defining B_4_C blade slit system. Two sets of beam clean-up slits and a beam diagnostic screen immediately precede a vacuum chamber containing mirror optics of the 100 nm-scale focusing system.

Downstream of the 100 nm-scale optical chamber, a differential pumping system is installed to support sample-injection compatible vacuum conditions in the X-ray beam–sample interaction region. The interaction region comprises a large vacuum chamber ∼600 mm × 600 mm × 800 mm (w × h × l), with infrastructure for several sample delivery methods, pump-laser in-coupling, imaging and diagnostics. The 100 nm-scale optical system and interaction chamber breadboard share a common granite support, which is designed to minimize any effect of vibration on the X-ray optical alignment of the 100 nm-scale beam.

The centralized SASE1 pump–laser system (Palmer *et al.*, 2019 ▸) is installed in an adjacent hutch and supplies the SPB/SFX instrument laser hutch with optical laser light at a repetition rate matched to that of the EuXFEL. Additional frequency conversion, beam conditioning and delay adjustment are implemented in the instrument laser hutch prior to delivery into the experiment hutch and interaction region.

The principal X-ray detector for the SPB/SFX instrument, the AGIPD 1 megapixel, is mounted into a carriage mated directly to the downstream flange of the interaction region chamber. The interaction region chamber and downstream devices including AGIPD are mounted onto a common rail system – the component support structure (CSS) – designed to move in order to track the X-ray beam trajectory, while preserving the alignment of downstream components. The detector carriage can be moved longitudinally along the rail, supporting sample–detector distances between 120 mm and 6000 mm.

A diagnostics screen and instrument beamstop occupy the downstream end of the CSS and are currently the final components of the SPB/SFX instrument, pending installation and commissioning of a second interaction region.

### 4.1. Optics and beam conditioning   

#### 4.1.1. Compound refractive lenses: initial focusing optics   

**Figure 3:**
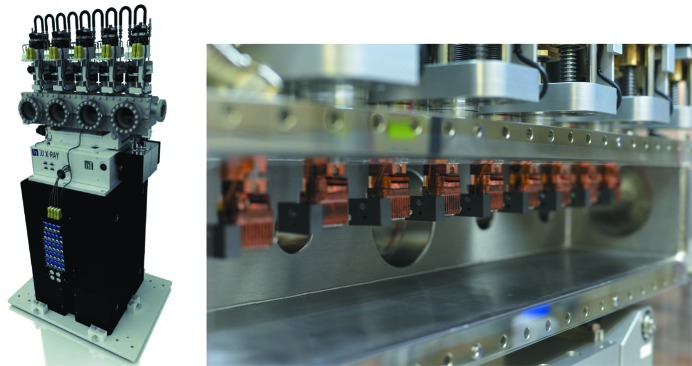
CAD-model of the JJ X-ray transfocator unit (left) and photograph of the lens cassettes inside the chamber installed in the XTD9 tunnel (right).

For the initial operation of the instrument during early user experiments (September 2017 to June 2018), focusing optics based on beryllium compound refractive lenses (CRLs) were installed to focus the beam to the X-ray–sample interaction point. The lenses are housed in a dedicated transfocator containing ten arms, each capable of holding up to ten lenses (JJ X-ray AS, Denmark). An image of the transfocator is shown in Fig. 3 ▸. The installation position at the end of the XTD9 tunnel and the long source-to-transfocator (∼888 m) and transfocator-to-focus (∼32 m) distances set the parameters of the lenses required.

To be able to focus over such long distances, lenses with large radii of curvature are required. Given the initial incoming X-ray beam parameters and minimal required energy range, 30 lenses with radii of curvature 5.8 mm and aperture 3 mm with beryllium grade IS50 (purchased from RXOPTICS GmbH & Co. KG, Germany) were installed, the largest radius of curvature available at the time of purchase.

**Figure 4:**
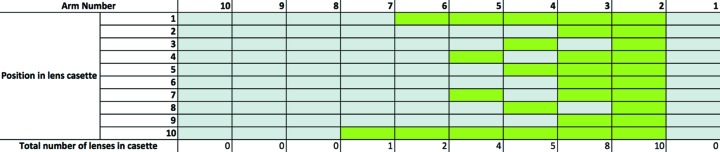
Arrangement of the beryllium lenses in the tranfocator unit, where green colour indicates in which position within the lens cassette the lens is placed. The arrangement is such that differing numbers of lenses can be placed into the beam by moving arms in or out, allowing for the focusing of different photon energies to the same plane in the interaction region.

Lenses were mounted into the lens cassettes in a binary configuration to enable a minimum increment of one lens for any lens configuration, as shown in Fig. 4 ▸. With the limitations on radius of curvature, the addition or removal of one lens adjusts the focus position by more than 1 m, larger than the longitudinal movement range of the transfocator. Fine tuning of the focus position to match the sample interaction point was successfully achieved by adjustment of the undulator gap and hence shifting the photon energy.

An image of the CRL-focused beam interaction with a 20 µm-thick cerium-doped yttrium aluminium garnet crystal (YAG:Ce) (Crytur, Turnov, Czech Republic) produced an upper bound on the focus size of ∼16 µm. This is larger than modelling would suggest and could be due to a number of reasons. Possibilities include (i) the chromaticity of the beam being greater than expected, (ii) the XFEL source being different from that expected, or even (iii) non-optimal alignment of the lens units. In practice, this limitation has been addressed by the replacement of the CRLs as the temporary, primary focusing optic by a super-polished mirror focusing system described in the next section.

#### 4.1.2. Super-polished mirror focusing systems   

The SPB/SFX instrument has two independent KB super-polished elliptical-mirror-based systems, focusing to the micrometre scale and 100 nm scale, to meet the variable X-ray beam size requirements of the science targets discussed in the preceding section. KB mirror systems were chosen as they are highly transmissive, achromatic and expected to survive the pulse train structure of the EuXFEL and largely preserve the wavefront properties of the FEL beam, critical for coherent imaging-type experiments. Both systems are designed to focus the X-ray beam to a common focal plane at the upstream interaction region of the instrument (see Fig. 1 ▸). At the time of writing, the micrometre-scale system has recently been commissioned and used in a small number of experiments. The focused beam from these optics was initially imaged at 5 µm, and recently improved to better than 3 µm as imaged with a 3.7 µm-thick LYSO:Ce (Lu_1.8_Y_.2_SiO_5_:Ce) scintillator at the interaction point. Continued commissioning and optimization of this focal spot is ongoing.

The optics of the 100 nm-scale system have recently been installed, with commissioning and first experimental use expected for mid-2019. Details of the performance of both mirror optical systems will be the subject of future publications.

The SPB/SFX instrument occupies the central branch of the SASE1 undulator beamline, minimizing the number of optical elements between the source and instrument focusing optics. The source point, estimated to be in the second-to-last undulator cell, is 918 ± 2 m from the instrument common focal plane (Bean *et al.*, 2016 ▸). The first optical elements in the SASE1 beamline are two B_4_C-coated flat offset mirrors installed for radiation protection. The second mirror includes a bender to allow flatness correction or to produce an intermediate focus between the offset mirrors and instrument optics (Sinn *et al.*, 2012 ▸; Tschentscher *et al.*, 2017 ▸). The position of the KB optic systems between the undulator source and instrument focus is determined by geometrical optics arguments, accounting for the predicted source size, desired focal size and source-to-focus distance. Given the large source–focus distance and expected beam divergence, 1000 mm silicon substrate mirrors (with 950 mm super-polished length) are installed. To maximize the available aperture, the mirror optics are designed for relatively high incidence angles of 4 mrad in the micrometre-scale case and 3.5 mrad in the 100 nm-scale case, with resulting apertures of 3.8 mm and 3.3 mm, respectively (Bean *et al.*, 2016 ▸). All mirrors are coated with two stripes, B_4_C and ruthenium, each 50 nm thick, for good transmission across the 3–16 keV working energy range of the instrument. Test measurements suggest that the coatings will survive the operating conditions at the SPB/SFX instrument (Aquila *et al.*, 2015*b* ▸). Further details on the polishing and control specifications of the mirrors are given by Bean *et al.* (2016 ▸).

The micrometre-scale system is located in the optics hutch, centred 23.2 m from the upstream interaction region common focal plane and 894.8 m from the predicted SASE1 source point. The two KB ellipses are incorporated into a four-bounce mirror system, with two additional flat mirrors of the same polishing and coating specifications, resulting in a focused beam with parallel trajectory and small offset from the direct beam. The depth of focus of the micrometre-scale system is calculated to be ∼10 mm.

The 100 nm-scale system is located directly upstream of the interaction region, centred 2.75 m upstream of the common focal plane. The 100 nm system uses a traditional two-bounce KB mirror scheme resulting in a 7 mrad angular deviation of the 100 nm-scale focused beam with respect to the direct beam. The depth of focus of the 100 nm-scale system is ∼0.15 mm.

**Figure 5:**
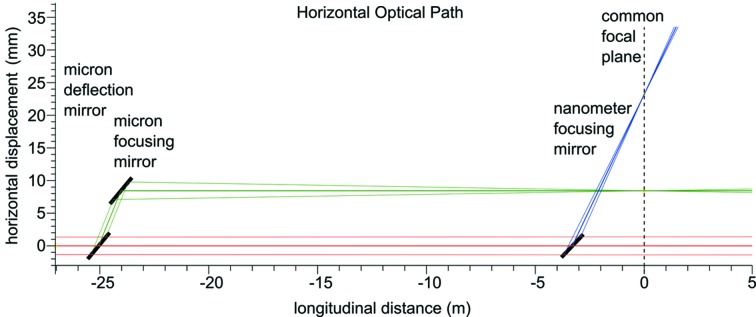
Representation of the horizontal optical layout of the SPB/SFX instrument. The incident beam from XTD9 is shown in red, with the 1 µm-scale system in green and the 100 nm-scale system in blue. The 0 m mark in the longitudinal distance denotes the common focal plane of the two systems. Figure adapted from Bean *et al.* (2016 ▸).

Representation of the horizontal and vertical optical paths of the direct, micrometre-scale and 100 nm-scale beams through the SPB/SFX instrument are shown in Figs. 5 ▸ and 6 ▸.

#### 4.1.3. Train picker   

A train-picker device is installed as the most upstream component of the SPB/SFX instrumentation in the XTD9 photon tunnel. This device is designed to pass individual (10 Hz) trains from the accelerator to the experiment either ‘on demand’ or synchronized with sample environment hardware. The key component is a notched disc which rotates to alternately block the beam or allow it to pass. The disc is composed of 2 mm B_4_C and 0.5 mm alloy of 98.5% tungsten with Ni and Fe. The train picker is intended for use only in low-power beam modes of 30 pulses per train or fewer.

#### 4.1.4. Beam conditioning   

To define the shape of the X-ray beam incident on the focusing optics, both the micrometre-scale and nano-scale focusing optics are accompanied by a slit system optimized for high incident beam power (JJ X-ray AS, Denmark). These so-called ‘power’ slits are equipped with four mechanically independent B_4_C ‘blades’ with a thickness of 75 mm along the beam direction. While B_4_C is among the materials with the lowest single-shot damage threshold for FEL radiation, it has a relatively long absorption length, especially at high X-ray energies. To more fully suppress radiation at higher energies, the B_4_C blocks are equipped with a 5 mm-thick sheet of tungsten on the downstream side. While the power slits are capable of blocking the unfocused incident beam at 10 Hz operation, they are not designed to withstand full pulse trains at 4.5 MHz with full beam power under all possible experimental conditions. In order to avoid overheating of the aperture-defining blocks under UHV conditions, each block is actively water-cooled.

A second set of slit systems is used to suppress scattering from the power slits and other sources of scattering such as the edges of the focusing mirrors. Permanent ‘cleanup’ slit installations (JJ X-ray AS, Denmark) are located between the micrometre-scale and the 100 nm-scale KB systems (see Fig. 1 ▸). Blade edges are composed of polished cylinders, fabricated in silicon nitride (low-*Z* option; JJ X-ray AS, Denmark) or wedge-shaped tantalum/tungsten (high-*Z* option; Xenocs SAS, France). Additional slit sets (customized development) can be installed inside the interaction region chamber to block remaining scatter close to the focus position. Polished cylinder-based blade edges of silicon nitride, tantalum/tungsten or germanium are available.

A solid attenuator array is installed to attenuate beam intensity in beam modes of up to tens of pulses per train. This compact device (JJ X-ray AS, Denmark) has four water-cooled arms, each equipped with six filters. Filters are primarily single crystal silicon, with thicknesses between 25 µm and 6.4 mm and polycrystalline synthetic diamond with thicknesses between 100 µm and 3.2 mm. Each arm can be inserted independently, giving a large range of possible attenuation options, roughly at least one combination in every magnitude interval of the transmission factors between 1 and <10^−12^ over the 3–16 keV operating energy range of the SPB/SFX instrument.

#### 4.1.5. Beam diagnostics   

To ensure an efficient alignment process of the complex X-ray optical focusing system and to maintain a high focus quality in the interaction region, 2D beam diagnostic devices are essential, especially for an FEL instrument (Tono *et al.*, 2011 ▸, 2013 ▸; Juranić *et al.*, 2018 ▸). For the first operation phase (before completion of the downstream interaction region), seven such devices are available at the SPB/SFX instrument, monitoring the beam at locations starting in the photon tunnel, before interaction with any focusing optics, down to the end of the instrument, immediately upstream of the instrument beam stop. To accommodate various beam sizes and positions at different locations within the instrument and also for different focusing schemes, two variations of the screen devices have been devised with different scintillator sizes (Type I and Type II, see below). Both screen types are non-transmissive. The series of screens allow monitoring of the beam position, trajectory, shape and internal structure.

The diagnostic screens, designed and built in-house, are based on flat scintillating screens with a standard thickness of 100 µm, made of single-crystal cerium-doped yttrium aluminium garnet (YAG:Ce) (Crytur, Turnov, Czech Republic). Scintillators are placed at 90° with respect to the X-ray optical axis, and an optically flat, λ/10, Al-coated fused silica mirror at 45° incidence angle is used to reflect the scintillation light through a high-grade anti-reflection-coated optical viewport, with flatness specification in the central region of λ/8, into an objective outside the vacuum chamber. Scintillators and mirrors are mounted to an aluminium frame inserted into the beam using a linear manipulator. Each Type I aluminium frame has four possible positions for scintillators, and each Type II has two, minimizing maintenance time in case a scintillator is damaged by the FEL beam. The additional mounting positions also allow the installation of additional diagnostic probes, such as area diodes.

**Figure 7:**
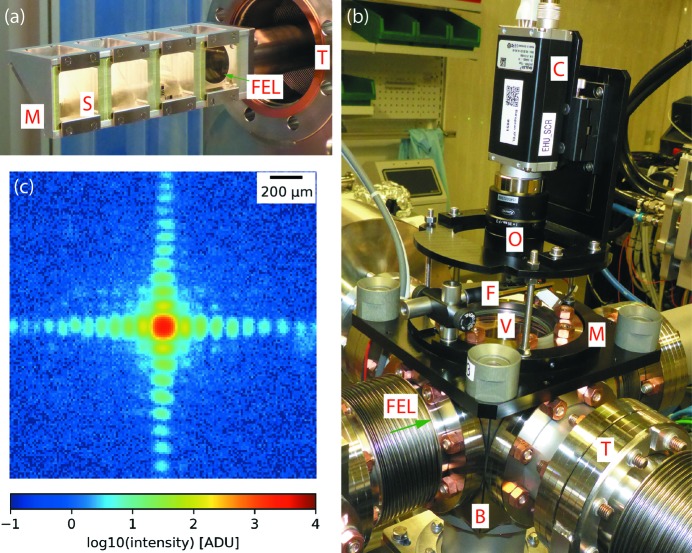
Overview of a beam diagnostic device at the SPB/SFX instrument. (*a*) UHV-compatible mounting block for four scintillators (S) and optical mirrors (M). (*b*) Overview of a Type I diagnostic device with a standard DN63CF cube as its basic building block (B). The three-point mount (M) for camera (C) and optics (O) can be removed without breaking the vacuum inside the cube for maintenance and baking. The scintillators are inserted into the beam via a motorized translation (T). An optical filter (F) is used to block transmission of the blue light from the reference laser which causes fluorescence emission from the scintillator just like the X-ray beam and thus can be visualized in the same way. (*c*) Illustrative example output from a Type I diagnostic device: diffraction from an instrument slit system closed to a gap of a few tens of micrometres in the horizontal and vertical direction, as imaged in a plane more than 22 m downstream at a photon energy of 9.3 keV. Only a very small fraction from the much larger incident beam was selected here. The image is a background-corrected average of 647 single-FEL-pulse images.

The main purpose of the diagnostic screens is to locate the beam centroid position in a relatively large field of view and are hence not optimized for high spatial resolution. While for Type I devices the resolution is limited by the pixel pitch of the sensor (effective pixel pitch ∼13.1 µm), for Type II devices it is limited by the objective’s object-side Rayleigh resolution (at maximum zoom around 9.5 µm, with an effective pixel pitch of about 3.0 µm). Installing higher-resolution optics for special applications is possible without opening up vacuum connections and only limited by the minimum working distance set by the distance between the scintillating screen and the viewport (∼93 mm for Type I devices and ∼119 mm for Type II devices). An image of a diagnostic screen and scintillator carriage is shown in Fig. 7 ▸ alongside a demonstration image.

#### 4.1.6. Instrument beam stop   

The instrument beam stop (IBS) is the most downstream component of the SPB/SFX instrument. Its main purpose is to attenuate and stop the FEL beam. The attenuating components are made from low-*Z* materials – diamond, B_4_C, and aluminium – with a large single-shot damage threshold.

**Figure 8:**
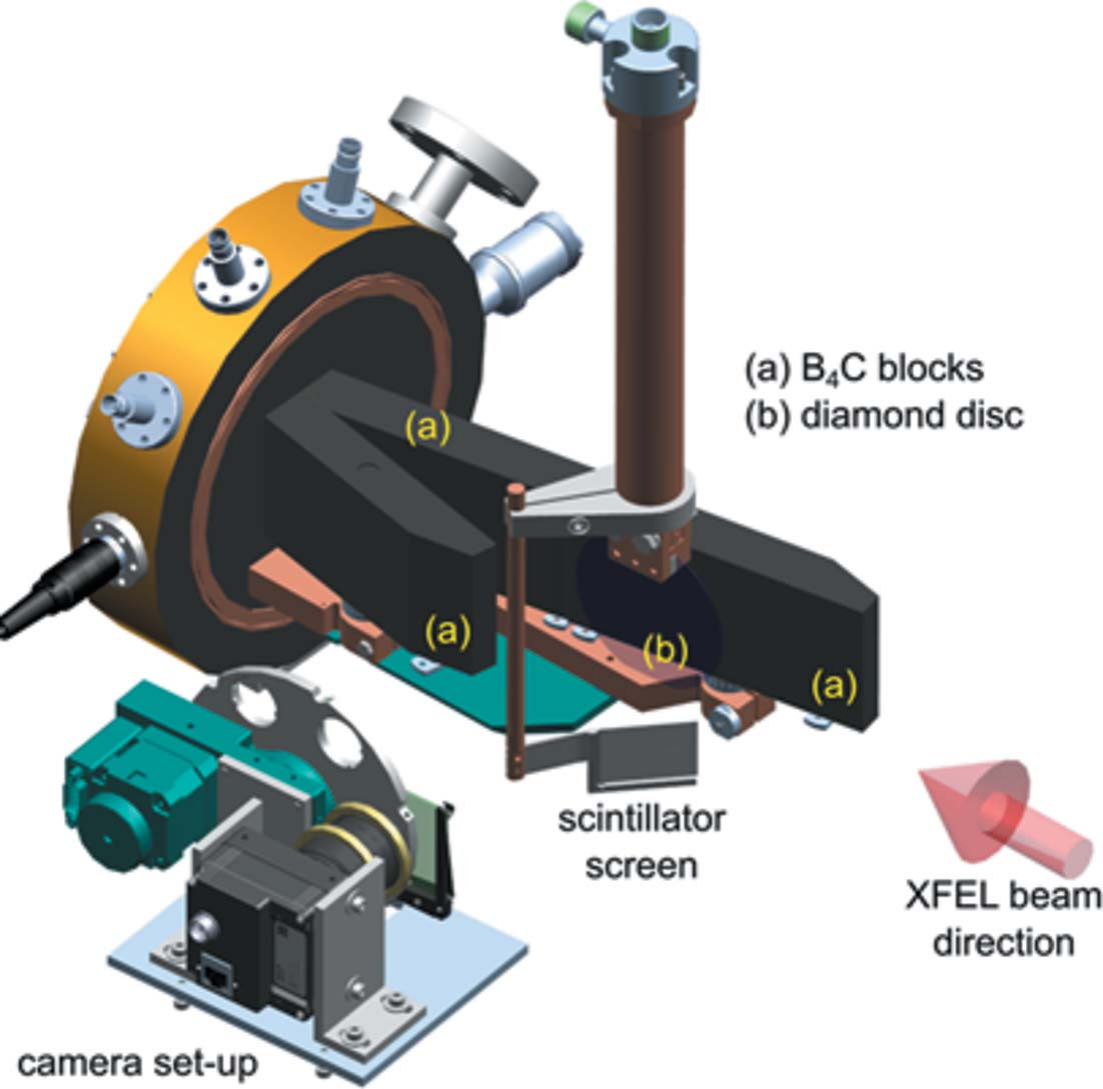
Schematic of the core components in the instrument beam stop (IBS). While a large fraction of the beam is attenuated by B_4_C blocks (*a*) arranged in a V-shaped layout, the initial material to attenuate the X-ray beam is a diamond disc at an incidence angle of about 2°. A camera setup is used to monitor the fluorescence signal caused by the X-ray beam incident on the diamond.

The main attenuating components are three B_4_C blocks that are arranged in an asymmetric V-shape, so that the X-ray incidence angle is about 10° with respect to the surface (see Fig. 8 ▸). Each B_4_C block has a heat sink terminal brazed to it, which is clamped in a water-cooled copper baseplate. All heat-sink terminals are electrically isolated from the B_4_C using sapphire discs, which makes it possible to read out an X-ray-induced photocurrent from each absorber as a diagnostic of beam intensity.

B_4_C is known to be among the most robust low-*Z* materials commonly used for the protection of X-ray components. Nevertheless, in-house front-end module (FEM) simulations indicate that even B_4_C would not withstand the full pulse train of the EuXFEL with the expected minimal beam size at the IBS position. The reason is high mechanical stress resulting from the thermal expansion of the B_4_C, following illumination. Therefore, the first component in the IBS that faces the direct X-ray beam is a diamond disc (diameter: 800 mm; thickness: 500 µm), which is composed of alternating layers of microcrystalline (thickness: 50 µm) and nanocrystalline (thickness: 5 µm) diamond (Diamond Materials GmbH, Freiburg, Germany). This composition was chosen to mitigate the risk of strong Bragg reflections from larger crystal grains, while maintaining the good thermal conduction properties of microcrystalline diamond. An incidence angle of about 2° further enhances heat distribution so that the disc can withstand the full pulse train, even in the worst case scenario. The diamond disc is clamped in a water-cooled copper arm. To monitor the X-ray beam during an experiment, fluorescence from this boron-doped diamond can be imaged using an out-of-vacuum camera setup (see Fig. 8 ▸).

### 4.2. Vacuum scheme   

The vacuum scheme of the SPB/SFX instrument is designed to provide appropriate conditions for instrumentation with differing vacuum requirements based on their position. Due to the potential power of the pulse train from the SASE1 undulators, and the desire to minimize background scattering from gas or windows, SPB/SFX is a windowless instrument from accelerator to undulator to sample injection to beamstop. Primary beam conditioning components and the KB mirror optical systems require UHV conditions (better than 1 × 10^−8^ mbar), while a higher pressure must be tolerated in the sample–X-ray interaction regions due to liquid- and gas-based sample injection. The upstream sections containing the conditioning and optical components are pumped using ion pumps and the downstream sections of the sample interaction regions and detectors are pumped with high-throughput turbomolecular pumps.

In order to maintain the UHV vacuum of the 100 nm-scale KB optics, a differential pumping system is installed between the optics vacuum tank and the interaction region chamber. The system is a three-stage differential pump design based on a series of pre-aligned straws with internal diameters of 3 mm mounted between sealed chambers, which are individually pumped using turbomolecular pumps. The narrow internal diameter of the connecting straws maintains a differential of approximately five decades between the interaction region chamber and the 100 nm-scale optics chamber. The differential system is mounted onto a four-axis stage – providing vertical, horizontal, pitch and yaw movements – to accommodate the differing trajectories of the 1 µm-scale, 100 nm-scale and direct X-ray beams.

Pumping of the X-ray–sample interaction region is provided by turbomolecular pumps attached to the chamber. The primary chamber pump is a 2300 L s^−1^ magnetically levitated pump, chosen to reduce vibrations transmitted to the chamber and, due to the continuous rotation axis adjustment, an anticipated improved performance under variable gas loads and potential particle contamination. An additional turbomolecular pump is connected to a port directly underneath the sample delivery mechanism ‘catcher’. This catcher provides a seal between the sample delivery and pump, establishing a differential vacuum, which allows the safe operation of the 2D X-ray detector (with high voltage and cooling systems interlocked to a pressure better than 1 × 10^−4^ mbar).

The turbomolecular pumps of the differential pumping system, interaction chamber and downstream sections of the instrument are supported with a distributed rough vacuum system. The outputs of the turbomolecular pumps are connected, via DN100 ISO-K standard aluminium tubing, to Ebara EV-S200P multi-stage dry roots pumps located in a pump room well separated from the experiment hutch to reduce noise and vibration. The ‘dirty’ rough vacuum of the interaction region and the ‘clean’ rough vacuum of the differential pump, detector and other downstream components are separated to avoid contamination, each having dedicated tubing and roots pump. A rough vacuum level of ∼5 × 10^−2^ mbar is maintained at the exhaust of the interaction chamber turbo pumps during sample injection.

### 4.3. Sample environment   

The SPB/SFX instrument deploys three main classes of sample delivery: (i) liquid jets for delivering (primarily) small crystals to the X-ray FEL beam for serial crystallography, (ii) focused aerosol beams for delivering (primarily) non-crystalline particles to the X-ray FEL beam for single particle imaging, and (iii) samples arranged on fixed targets, which may be crystalline or non-crystalline. All three sample delivery methods are supported in the upstream interaction region (IRU) of the SPB/SFX instrument. Additional, downstream interaction regions (IRD) are planned as extensions of the baseline instrumentation and are outlined in Section 4.6.

#### 4.3.1. Liquid jet sample delivery   

Liquid jets are the principal sample delivery method for serial femtosecond crystallography experiments. The liquid jet delivery system at the SPB/SFX instrument is designed to maintain compatibility with established jet delivery systems developed at other FEL facilities while allowing flexibility in nozzle design. More details are given by Schulz *et al.* (2019 ▸).

Sample delivery nozzles are mounted at the end of a hollow rod, inserted via a load lock to position the nozzle above the X-ray focus position. Currently, two configurations are available: a 1/2-inch outer-diameter rod and a 25 mm version suitable for more complicated nozzle designs.

The nozzle rod mates to a catcher centred around the interaction region. The catcher includes apertures for the FEL beam, a 100° solid angle diffraction exit cone, as well as windows for microscopes and optical laser coupling. The catcher restricts the majority of sample residue to a small easy-to-clean volume and provides differential pumping of the liquid and gas loads. Both nozzle rod and catcher assembly are modular in design for easy upgrade.

The most frequently used liquid jet devices are gas dynamic virtual nozzles (GDVNs) (DePonte *et al.*, 2008 ▸). GDVNs compress a liquid sample jet from a 50–100 µm capillary to a few micrometres diameter using a surrounding gas stream. These nozzles can provide fast jets with velocities over 80 m s^−1^ (Grünbein *et al.*, 2018*b* ▸), recently shown to successfully deliver sample jets compatible with the current operational pulse train structure of the FEL beam at SPB/SFX (Grünbein *et al.*, 2018*a* ▸; Wiedorn *et al.*, 2018 ▸).

While not ideally suited to the pulse train structure of the EuXFEL, high-viscosity extrusion jet sample delivery (Weierstall *et al.*, 2014 ▸) can be installed into the nozzle rod and catcher infrastructure. Initial experiments with this delivery method have been limited to a single pulse per train, at a train rate of 10 Hz.

#### 4.3.2. Aerosol sample delivery   

Aerosol sample delivery is expected to be the primary method for delivering single particles into the X-ray beam for imaging experiments at the SPB/SFX instrument. The small scattering cross section and non-crystalline nature of single particles require that the scattering background is reduced to a minimum. In aerosol-based sample delivery, the aim is to isolate the sample from any surrounding liquid, removing scattering from the delivery medium that would otherwise overwhelm the weak sample scattering signal.

An aerosol of (sub-)micrometre-sized droplets, each containing ideally one sample particle, is generated from a nozzle in a chamber at the entrance to an aerodynamic lens stack. The droplets in this aerosol mist evaporate, in principle leaving behind isolated sample particles that are funnelled into an aerodynamic lens (Hantke *et al.*, 2018 ▸). Within the aerodynamic lens, the particle flow is focused into a narrow beam that intersects the X-ray beam at the exit of the lens stack.

**Figure 9:**
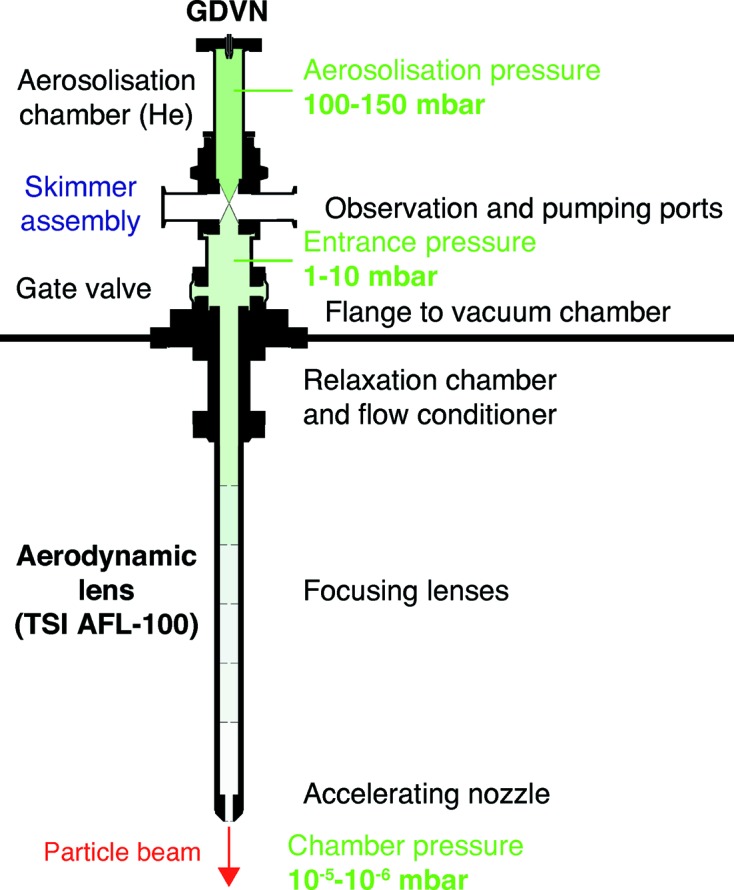
Schematic of an aerosol injector. The droplets are formed inside the aerosolization chamber from the Rayleigh instability in a thin liquid jet originating from a flow focusing nozzle. The gas necessary for the flow focusing is further used both to create an atmosphere where the droplets are able to evaporate and to provide the stream-lines inside the aerodynamic lens used to focus the particles into a narrow beam. Between the aerosolization chamber and the lens stack, a nozzle-skimmer stage is inserted to limit the gas load inside the lens stack. Adapted from Hantke *et al.* (2018 ▸).

With the aerodynamic lens stack available at SPB/SFX (see Fig. 9 ▸), efficient delivery of particles ranging from 30 to 3000 nm in diameter is possible. The nozzle-to-interaction particle transmission varies with both particle size and gas flow, and can reach >70% (Hantke *et al.*, 2014 ▸) for particles of a few hundred nanometres in diameter. The exit velocity also depends on particle diameter and gas flow, with sub-100 nm particles reaching 200 m s^−1^, while micrometre-sized particles travel slower at approximately 20 m s^−1^ (Hantke *et al.*, 2018 ▸).

Potential complications of limited liquid jet speeds in the high peak repetition rate FEL beam, such as sample replacement and jet disruption (Stan *et al.*, 2016 ▸), can be alleviated with aerosol sample delivery, where no surrounding liquid is present.

The lack of surrounding liquid also enables the use of ion time-of-flight spectroscopy as a potential means to provide detector veto signals within a single pulse train (Andreasson *et al.*, 2014 ▸; Pietrini *et al.*, 2018 ▸). These features make aerosol sample delivery an intriguing possibility for delivering crystalline samples into the X-ray beam (Awel *et al.*, 2018 ▸).

### 4.4. Detectors: overview and present installation   

Crystallographic and single-particle experiments rely on high-performance 2D X-ray photon detectors. Suitable detectors must be capable of measuring fully integrated 2D diffraction patterns from single tens of femtosecond X-ray exposures. They must have a high dynamic range to capture intense Bragg peaks as well as low scattering signals from non-crystalline samples, all at the maximum 4.5 MHz pulse rate of the EuXFEL (Giewekemeyer *et al.*, 2013 ▸).

One of the area detectors developed specifically to capture diffraction patterns at the unique time structure of X-ray pulses produced by the EuXFEL is the Adaptive Gain Integrating Pixel Detector (AGIPD) (Allahgholi *et al.*, 2016 ▸; Mezza *et al.*, 2016 ▸; Allahgholi *et al.*, 2019 ▸). Two instances of AGIPD will ultimately be deployed at SPB/SFX, a 1 megapixel (1Mpx) version, already in use at the upstream interaction region since September 2017, and a 4 megapixel (4Mpx) version planned for the downstream interaction region. A Jungfrau 4Mpx detector (Jungmann-Smith *et al.*, 2016 ▸) will be available for use as a secondary detector at experiments at the downstream interaction region of SPB/SFX.

The specifications of the AGIPD 1Mpx are described below, and the AGIPD 4Mpx and Jungfrau are outlined later in the description of the future downstream interaction region. The mechanical setups of each AGIPD implementation and the Jungfrau detector feature motorized longitudinal motion in order to vary the sample–detector distance.

#### 4.4.1. The Adaptive Gain Integrating Pixel Detector   

AGIPD is a hybrid pixel array, silicon sensor-based detector with 200 µm × 200 µm pixels (Allahgholi *et al.*, 2016 ▸). It is an integrating detector (rather than counting) and hence able to acquire full diffraction patterns from single EuXFEL shots, which is essential for the diffraction-before-destruction approach of serial femtosecond crystallography.

The smallest unit of AGIPD is a single FEM, consisting of 128 × 512 pixels, an active area of ∼26 mm × 103 mm. Multiple FEMs can be assembled to form a large 2D area detector. Each FEM is constructed from a 500 µm-thick silicon sensor, giving a high absorption efficiency over the 3–16 keV photon energy operation range at SPB/SFX. The sensor surface facing the interaction region is coated with a 500 nm layer of aluminium to prevent the absorption of optical light photons, especially important for maintaining a low background level in optical-pump-type experiments. The sensor is bump-bonded to an application-specific integrated circuit (ASIC) for detection of the absorbed X-ray photons.

The ASIC is optimized for the high dynamic range, high peak repetition rate demands of experiments at SPB/SFX. It features a radiation-hard electronic design to ensure several years of operation. High dynamic range is achieved via ‘adaptive gain’ switching of individual pixels between three gain levels in adaptation to the incident signal. At a photon energy of ∼12 keV, signal levels of 10^4^–10^5^ photons pixel^−1^ pulse^−1^ (*i.e.* 10^4^ at 12 keV, more at lower energies) can be recorded in low gain mode, alongside single-photon sensitivity in high gain mode.

Data acquisition at up to 4.5 MHz is achieved by storing signal values in on-pixel analogue memory for the duration of the pulse-train, and read out and subsequently digitized during the idle time (99.4 ms) in between pulse trains. Up to 352 values per pulse train can be stored in the on-pixel memory, resulting in a maximum data rate from AGIPD of 3520 recorded images per second (Allahgholi *et al.*, 2016 ▸).

#### 4.4.2. AGIPD 1Mpx   

**Figure 10:**
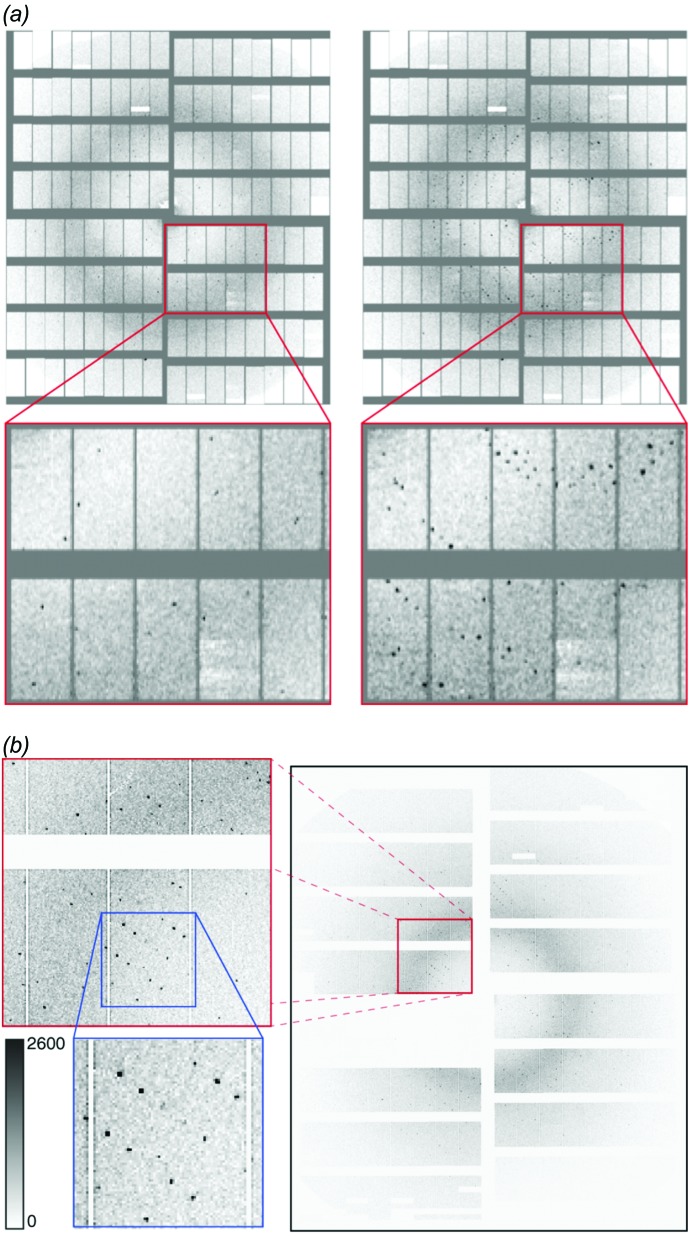
Example serial crystallography data taken using the AGIPD 1Mpx during early user experiments at SPB/SFX. Note the well defined Bragg peaks that span a large fraction of the detector’s dynamic range. This figure was originally published in (*a*) Grünbein *et al.* (2018*a* ▸) and (*b*) Wiedorn *et al.* (2018 ▸) and is licenced under a Creative Commons Attribution 4.0 International Licence.

The first produced AGIPD 1Mpx detector is installed at the upstream interaction of the SPB/SFX instrument and has been used for both crystallography and single-particle imaging-type experiments. Fig. 10 ▸ shows some examples of serial crystallography data taken to date. AGIPD 1Mpx comprises 16 FEMs, grouped into four quadrants centred around the X-ray beam, to form a 1024 × 1024 pixel, 1Mpx detector. Each quadrant is designed to be individually positioned around the X-ray beam such that the position, size and shape of the central aperture can be adjusted.

**Figure 11:**
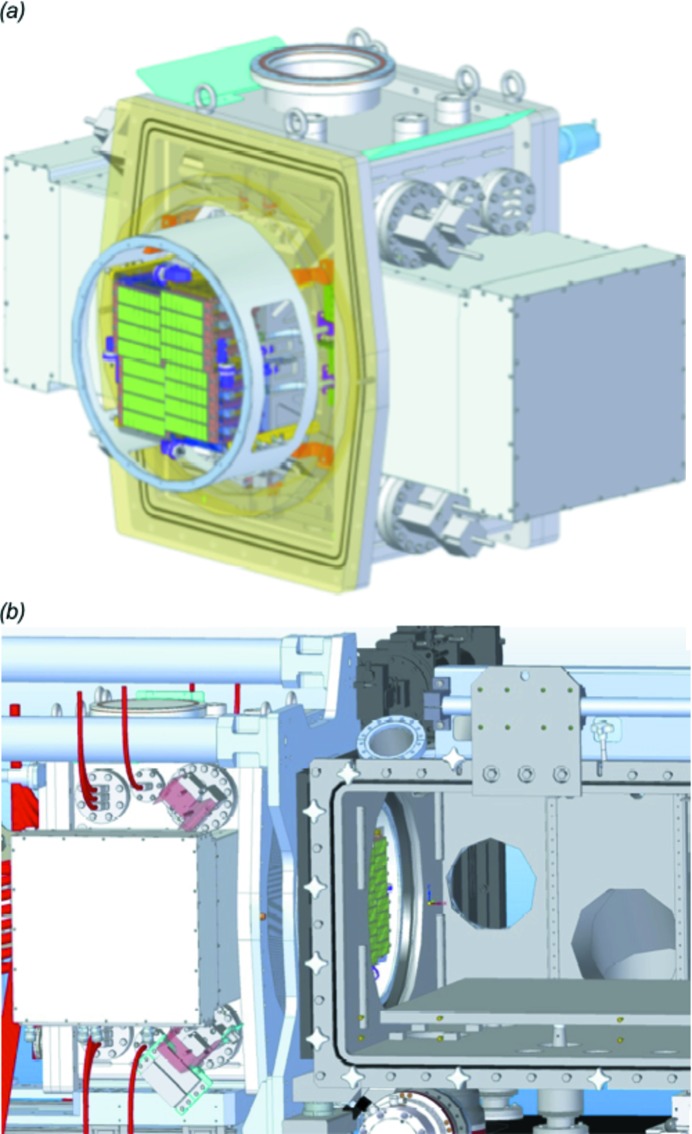
(*a*) Mechanical design of the AGIPD 1Mpx detector inside its vacuum tank. The detector consists of four movable quadrants, which are predominantly moved in an iris fashion to adjust the central hole size. Each quadrant in turn consists of four AGIPD FEMs. (*b*) CAD-model of the AGIPD in its most upstream position (sample-to-detector distance ∼120 mm). The compressed bellows can also be seen between the sample chamber and the AGIPD vacuum chamber. The sample chamber is deliberately shown empty so one can see the AGIPD sensors (in green) that detect the X-ray photons.

AGIPD 1Mpx is installed in a vacuum chamber mounted in a detector carriage with large-diameter bellows between the upstream and downstream flanges of the cage. An engineering model of AGIPD 1Mpx in its vacuum chamber is shown in Fig. 11 ▸. This construction provides up to 200 mm longitudinal movement of AGIPD without breaking vacuum and supports a minimum sample–sensor distance of 120 mm (2θ angle of ∼45° at the upper/lower edges). The carriage can either be mounted directly to the exit flange of the interaction region chamber or behind a modular flight tube for sample–sensor distances of up to 6 m.

### 4.5. Optical laser systems   

There are three optical laser systems relevant to operation of experiments at the SPB/SFX instrument. The first are the reference lasers, a simple setup for allowing alignment of components when the FEL beam is not present. The second are a collection of commercial nanosecond lasers, used for pump–probe timing where the dynamics of the sample are longer than nanoseconds. The third is a EuXFEL-developed central laser system (Palmer *et al.*, 2019 ▸) that offers femto­second and picosecond pulses at the repetition rate of the accelerator, suitable for pump–probe experiments on sample systems with nano-, pico- or femtosecond timescale dynamics.

#### 4.5.1. Reference lasers   

A preliminary alignment of the instrument requires a reference laser, collinear with the X-ray beam. A reference laser can be used to align components and apertures without the X-ray beam, minimizing installation effort when changing experimental environments and reducing the risk to X-ray sensitive components.

The specific needs of the SPB/SFX instrument require ±5 mm horizontal and vertical positioning to match the 1 µm-scale focused beam and 100 nm-scale focused beam trajectories, variable longitudinal focus position along the instrument, microradian-order angular precision, and remote control of in-coupling.

The reference lasers (Wavespectrum WSLS-445-001m-4) are installed in locations at the upstream ends of the optics and experiment hutches, upstream of the respective mirror focusing systems (see Figs. 1 ▸ and 2 ▸). Each reference-laser beam can be introduced into the X-ray vacuum beam path by an in-coupling optical mirror that may be remotely driven into a fixed kinematic mount to guarantee positional reproducibility.

The 445 nm wavelength laser excites the Ce:YAG scintillators of the beam diagnostic screens, and the resulting fluorescence can be viewed with the screen cameras to establish target positions for the X-ray beam on each screen and ensure a clear path through the interaction region equipment and detectors.

#### 4.5.2. Commercial nanosecond lasers for pump–probe experiments   

Three nanosecond optical parametric oscillators (OPOs), Opolette HE 355 LD UV from Opotek (Carlsbad, CA, USA), are available at SPB/SFX. These OPOs can provide 4–7 ns pulses of mJ order, continuously tunable in the range 210–2400 nm, relevant for a number of biological systems with microsecond or even millisecond response times. These lasers are, however, limited to 10 Hz operation. Up to three OPOs can be used concurrently for experiments that require multi-step excitation sequences.

#### 4.5.3. Optical femtosecond and picosecond lasers and their conditioning   

Optical pump–X-ray probe experiments require a synchronized optical laser as a pump source. The EuXFEL Optical Lasers group provides 800 nm, 15–300 fs near-transform-limited pulses, with central wavelength tuneable across a 750–850 nm range and 1030 nm, 1, 400 ps (compressed, stretched, respectively) pulses to the SPB/SFX instrument laser hutch (ILH) (Palmer *et al.*, 2019 ▸), see Table 2 ▸.

Parameters of the two laser systems at various set points are summarized in Table 3 ▸, and detailed information has been given by Pergament *et al.* (2016 ▸). The ILH contains beam diagnostics, beam conditioning and delay stages to prepare an appropriate pump beam for biological samples. Additional optical components in the experiment hutch and chamber guide the laser beam to the X-ray–sample interaction point. The 800 nm femtosecond laser pulses are delivered negatively chirped to the ILH to compensate for dispersion introduced by the windows of the beam transport pipes and the sample chamber as well as the air path, ensuring optimal pulse duration at the sample position.

Harmonic generation – up to the third harmonic for the 800 nm system and fourth harmonic for the 1030 nm system – is provided in the experiment hutch to enable alternative pump wavelengths. An optical parametric amplifier, TOPAS Prime with NirUVis and NDFG units (Light Conversion, Vilnius, Lithuania), will be installed to provide continuous tunability of the femtosecond laser system, covering the wavelength range 240–15000 nm. Specific transport optics can then be installed to support a given subset of that wavelength range for any given experiment.

**Figure 12:**
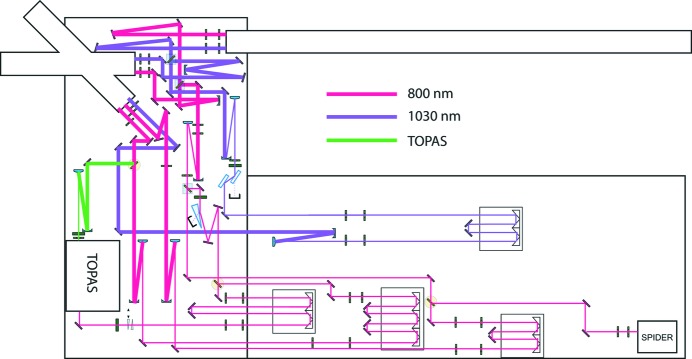
Laser optics layout in the SPB/SFX instrument laser hutch (ILH) for the upstream interaction region (IRU). The optics table size is 1500 mm × 3000 mm (h × w) (left area) and 3600 mm × 1500 mm (h × w) (right area). Beam from the SASE1 common pump laser installed in the central laser hutch (CLH) enters from the left. The optical path to IRU exits top left, and transport of the common pump laser to the optical table for the downstream interaction region (IRD) is shown at the top.

Fig. 12 ▸ shows the optical layout in the SPB/SFX instrument laser hutch. The optical laser paths (800 nm, 1030 nm) have delay lines to control the pump-pulse time of arrival with respect to the X-ray pulse. All beam paths have a consistent 15 m length in the ILH.

All reflective mirrors are equipped with very low dispersion coatings appropriate for the respective wavelength operation regime. The mirrors and other optics require sufficient damage thresholds and low group delay dispersion (GDD) to maintain performance for high peak power and high intensities at high-repetition-rate conditions. For the shortest 15 fs pulses, high specification coated mirrors are in use [*e.g.* Ag + multilayer HRs (45°, 680–940 nm) > 99.5% |GDD| <10 fs^2^ and HR 45° Rp > 99.97% Tp ≤ 0.02% for 1030 nm high-power ps-pulses from Layertec (Mellingen, Germany)].

Different wavelengths require different mirror solutions. Mirrors with silver-based coatings can be used in a wavelength range from 440 nm to the near-infrared. For other cases, which require high-power damage threshold or low GDD in the UV region, customized dielectric coatings are used.

Air humidity and temperature stability are key for consistent delivery of ultrashort pulses. For example, a 10% relative humidity change will alter the refractive index of air by 0.07 p.p.m. (Telada, 2009 ▸), introducing a 5 fs timing jitter for a path length of 20 m (approximate total path length to the sample). A 1 K temperature change can induce a 660 fs arrival time offset for the same 20 m path length (thermal expansion coefficient of type SUS410 stainless steel 9.9 × 10^−6^ K^−1^). To minimize these effects, a high-precision air-conditioning system is installed in the central laser hutch and ILH, specified to a 21 ± 0.1 K temperature range and 50 ± 1% humidity. Recently, very first measurements of the arrival time jitter between X-ray and optical pulses have been made (Kirkwood *et al.*, 2019 ▸). The measured jitter is expected to improve significantly in the near future.

### 4.6. Downstream interaction region: overview   

As the popularity of serial crystallography increases, the opportunity for an additional experimental region is advantageous to improve the efficiency and throughput of the SPB/SFX instrument as a whole. The downstream interaction region (IRD) aims to maximize the use of the X-ray pulses of the EuXFEL by refocusing and reusing the direct beam after it has passed through the upstream interaction region (IRU) to a second experimental setup (Boutet *et al.*, 2015 ▸; Hunter *et al.*, 2016 ▸). Refocusing the used beam with CRLs mounted on the CSS, it is intended to enable two experiments to be performed simultaneously – one upstream and one downstream. This additional instrumentation has been contributed by the SFX user consortium (SFX User Consortium, 2013 ▸).

The downstream interaction region, in practice, comprises two separate (and mutually exclusive) interaction regions. One will be operable in atmosphere or a helium environment (IRDa), while the other will operate in vacuum (IRDb).

IRDa gives users increased flexibility in their choice of sample delivery; for example, allowing studies for thermal- and pressure-sensitive samples. Additional scope for developments of novel concepts for sample delivery are also possible (without the complication of vacuum compatibility) as well as accessing longer timescales where the sample may freeze in vacuum and for more complex multi-stage optical pumping to access intermediate states in multi-stage reaction cycles.

As reliable and reproducible data collection is a prerequisite, standardized sample environments will be available, such as the Roadrunner system (Roedig *et al.*, 2017 ▸) (version III, in atmosphere) with an option for nozzle mounting, which will allow for crystal injection in liquids of viscous media and fixed target data collection. Crystallography data is planned to be collected at atmospheric pressure using Jungfrau detector modules. To enable experiments at atmospheric pressure, safe outcoupling of the beam downstream of the CRL refocusing optics will use an in-house-designed sonic delay line to protect the upstream components in case of failure of the outcoupling diamond window. Commissioning of IRDa is planned for 2019 with more regular operation expected from early 2020.

IRDb consists of an in-vacuum sample environment and a 4 megapixel AGIPD for detection and is presently planned for an early 2020 installation.

#### 4.6.1. AGIPD 4Mpx   

The in-vacuum downstream interaction region of SPB/SFX will be equipped with a 4 megapixel AGIPD. The mechanical design is optimized for crystallographic experiments to match the primary scientific scope of the downstream interaction region. In addition to the primary use as the principal detector of the downstream interaction region, AGIPD 4Mpx can serve as a second detection plane for upstream interaction region experiments requiring recording of very small angle signal. The AGIPD 4Mpx sensor plane is located ∼10 m downstream of the IRU focus position.

AGIPD 4Mpx consists of 56 AGIPD FEMs (3.7 megapixel) of the same type as AGIPD 1Mpx, arranged in two halves of 28 FEMs each. The detector halves are vertically offset by half of the FEM pitch to ensure continuous reciprocal space coverage for samples with a delivery orientation preference (Redecke *et al.*, 2013 ▸). The total detection plane area is larger than 400 mm × 400 mm, requiring an 800 mm-diameter entrance flange.

The detector halves can be independently positioned, both transversely and longitudinally. Each half can move horizontally up to 50 mm to control the position and size of the central slot, with a maximum separation of 100 mm. Sample–sensor distances in the range of ∼150 mm (2θ angle of ∼60° at the upper/lower edges) to 550 mm will be achievable. In contrast to AGIPD 1Mpx, most of the electronics of AGIPD 4Mpx, including digitizers and FPGAs, will be installed in-vacuum, directly attached to the output of each FEM.

The AGIPD 4Mpx detector of the IRD is also planned to be used as a second detection plane for samples injected in the upstream interaction region (IRU) with the two regions connected by a long evacuated flight tube to facilitate single particle imaging, solution scattering measurements, and other experiments requiring very low angle (scattering/diffraction) data to be collected.

#### 4.6.2. Jungfrau   

In the in-atmosphere downstream interaction region, SPB/SFX will integrate a Jungfrau 4Mpx detector (Jungmann-Smith *et al.*, 2016 ▸). The Jungfrau detector has many similarities with AGIPD. Jungfrau is an integrating hybrid-pixel array detector, based on 512 × 1024 pixel FEMs using a 320 µm-thick silicon sensor. The interaction-region facing surface of the sensor is coated in aluminium to prevent the absorption of optical light. High dynamic range is realized with gain switching. At a photon energy of 12 keV, signal levels of 10^4^ photons pixel^−1^ pulse^−1^ at 12 keV can be recorded in low gain mode with single-photon sensitivity in high gain mode in an adjacent pixel.

Data acquisition at possibly up to megahertz rates is achieved by storing signal values in on-pixel analogue memory for the duration of the pulse train, and read out and subsequently digitized during the idle time (99.4 ms) in between pulse trains. Sixteen values per pulse train can be stored in the on-pixel memory, resulting in a maximum data rate from Jungfrau of 160 recorded images per second (Jungmann-Smith *et al.*, 2016 ▸).

In comparison with AGIPD, Jungfrau has a smaller pixel size of 75 µm × 75 µm and reduced electronic noise, leading to enhanced single-photon sensitivity at the low end of the SPB/SFX photon-energy operation range. Prior to full integration and commissioning of the SPB/SFX downstream interaction region, a preliminary implementation of Jungfrau at SPB/SFX will consist of four FEMs (2 megapixel), arranged in two independently movable halves. An upgrade to 4 megapixel will be completed upon receipt of additional modules. This detector will initially be housed in a manner compatible with a helium sample environment (see Section 4.6 with later upgrades to a vacuum housing possible).

## 5. Conclusions   

The SPB/SFX instrument has been described, including its early operation optical system, sample delivery systems, detection systems and basic diagnostics. Importantly, the first scientific results from user experiments at the EuXFEL (Grünbein *et al.*, 2018*a* ▸; Wiedorn *et al.*, 2018 ▸) have been published demonstrating that serial crystallography is indeed possible at MHz rates. This, in turn, allows for unprecedented data rates, which supports high-throughput experiments, finer time steps in time-resolved work, or both. The possibilities of more than an order of magnitude faster data collection than any other similar source are just being explored.

Beyond this advantage of higher repetition rate, at present, a whole suite of additional instrumentation is being installed that will provide further capabilities to EuXFEL users in the not-too-distant future. The capabilities include detectors with more pixels, alternate sample delivery mechanisms, additional diagnostics, and more. With both MHz repetition rates and a suite of instrumentation tailored to the needs of structural studies, the future promises to hold a number of exciting possibilities at the SPB/SFX instrument of the EuXFEL.

## Figures and Tables

**Figure 6:**
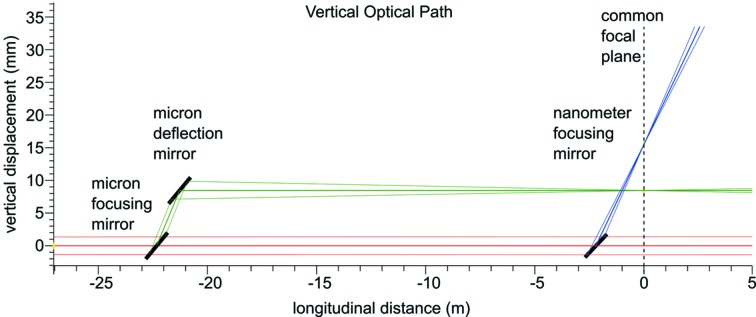
Representation of the vertical optical layout of the SPB/SFX instrument. The incident beam from XTD9 is shown in red, with the 1 µm-scale system in green and the 100 nm-scale system in blue. The 0 m mark in the longitudinal distance denotes the common focal plane of the two systems. Figure adapted from Bean *et al.* (2016 ▸).

**Table 1:** Summary of basic parameters of the the SPB/SFX instrument

Parameter	Value/range	Units
Photon energy	3–16	keV
Pulse energy (maximum)	∼1–5	mJ
Photons per pulse (maximum)	∼1–8	10^12^ photons
Focal spot size	∼100s, 1	nm, µm
Repetition rate	10 × 1350 (maximum at highest pulse energy)	s^−1^
	10 × 2700 (maximum at lower pulse energies)	
Pulse duration (range)	A few 100	fs
Detector pixel size (AGIPD)	200 × 200	µm
Upstream detector	AGIPD, four independent quadrants (4 × 512 × 512 pixels)	NA
Downstream detectors (to be installed in future)	AGIPD and Jungfrau, both 4 megapixel	NA
Single-photon sensitivity	Yes	NA
Detector dynamic range at 12 keV	>10^4^	photons
Detector frame rate AGIPD (burst)	4.5	MHz
Sample–detector distances	0.12 to ∼6	m
Sample delivery options	Liquid jet, aerosol jet, fixed targets	NA
Miscellanea	Pump–probe laser	NA

**Table 2:** Description of basic laser parameters The 800 nm source is tunable from 750 to 850 nm (longer than 15 fs).

Wavelength λ	800 nm	1030 nm
Pulse duration (FWHM)	15–300 fs (nearly Fourier-transform-limited)	<1 ps or 400 ps (chirped)

**Table 3:** Laser set points

Set point	Repetition rate (MHz)	E1030 nm (mJ)	E800 nm (mJ)
1	4.5	1	0.05
2	1.13	4	0.3
3	0.188	21	1.5
4	0.1	40	2.5
